# Confined nanospace for enhanced photocatalysis

**DOI:** 10.1093/nsr/nwab003

**Published:** 2021-01-08

**Authors:** Bao-Lian Su

**Affiliations:** State Key Laboratory of Advanced Technology for Materials Synthesis and Processing, Wuhan University of Technology, China; Laboratory of Inorganic Materials Chemistry (CMI), University of Namur, Belgium

The world today is very different from how it was. The consumption rate of fossil fuels compared with the available resources and the calamity of global warming frighten people. The entire planet is at risk, but these are our crises. Mankind is the cause and so mankind should find sustainable and green energy solutions to satisfy the needs of society.

Chemical reactions driven by solar energy can not only diminish consumption of fossil fuels and pollution from such consumption, but also achieve high selectivity through exact energy exchange between photons and electrons. Since the pioneering work on electrochemical photolysis of water in 1972, semiconductors and other light-absorbing materials such as plasmonic metals have been widely explored [1–3]. For a long time, scientists have focused on enhancing photocatalytic performance through optimization of the three crucial processes in photocatalysis: light harvesting, electron-hole separation, and surface redox reactions. With the development of nanotechnology, hollow nanostructures have been found to efficiently promote light absorption and charge separation [4]. Not only that, the confined nanospace in the hierarchically hollow structures can provide a specific microenvironment for the reactions and affect the mass transfer of substrate to achieve a maximized efficiency [5–7].

To demonstrate the vital role of confined nanospace, a recent cooperative research group led by Prof. Jian Liu from the Dalian Institute of Chemical Physics, Chinese Academy of Sciences and Prof. Jun Huang from the University of Sydney investigated the effect of hierarchically hollow-structured nano-photoreactors loaded with bimetallic catalysts at different positions for photocatalytic oxidation of cinnamyl alcohol [8]. Hierarchically hollow nanostructures can be obtained through a nicely designed spontaneous phase transformation of core-shell structured zeolitic imidazolate frameworks (ZIF)-8@SiO_2_ to hollow mesoporous zinc silica composites (HMZS) under mild hydrothermal conditions. The research traces the transformation process under different conditions via *semi-in situ* technique, and an ‘adhesive-contraction’ mechanism of hollow structure formation is proposed. Meanwhile, the synthesis strategy ensures controllability of the spatial location of active metals. The situation of AuPt bimetallic nanoparticles inside/on the surface (AuPt@HMZS and AuPt/HMZS) of the hollow structures depends on the order of metal loading and shell coating.

AuPt bimetallic nanoparticles inside/on the surface of the hollow structures exhibited a broader absorbance region under visible light than monometallic, through the influence of strong metal–metal interactions, as shown in Fig. 1a. Intriguingly, the adsorption edge of AuPt@HMZS displayed a red-shift compared with AuPt/HMZS. This phenomenon can be explained by multiple scattering of light inside, and the confined space of the hollow structure. It is clear that light absorption can be improved efficiently in a confined space, accompanied by heat management. The heat generated by light scattering and the photothermal effect of plasmonic AuPt nanoparticles is usually weakened via electron-photon scattering to reach the system equilibrium. However, heat loss can be slowed as the hollow nanoreactor serves as an insulating layer. As a result, the temperature in such a confined space where the reaction occurs will be higher than that of the external environment. The reaction rate of cinnamyl alcohol with AuPt nanoparticles confined inside the hollow structure (AuPt@HMZS) is 3.1 mmol/g after 5 h under visible light irradiation, which is superior to that of AuPt nanoparticles located at the surface of the hollow structure (AuPt/HMZS) with similar amounts of Au and Pt (Fig. 1b).

**Figure 1.**
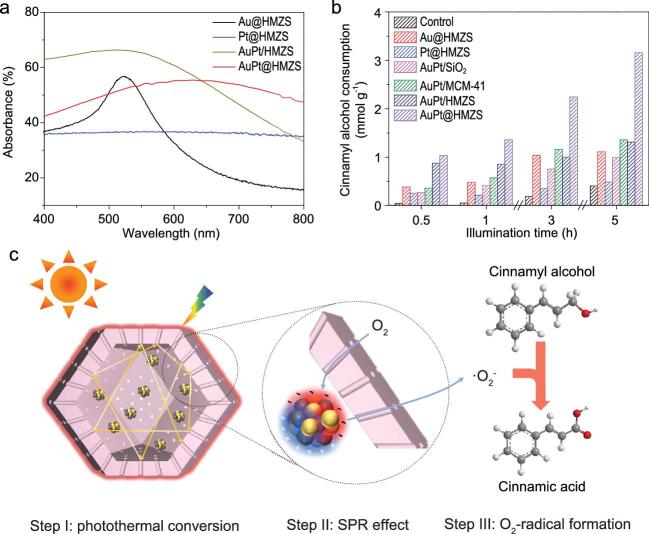
(a) UV–Vis spectra of AuPt@HMZS and other contrast catalysts. (b) Photocatalytic oxidation performance of AuPt@HMZS and other contrast catalysts to cinnamyl alcohol solution. (c) Proposed mechanism responsible for the photocatalytic oxidation of cinnamyl alcohol in the presence of AuPt@HMZS. Reproduced with permission [8], Copyright 2020, Oxford University Press.

The contribution of hot electrons to the reaction process was also investigated. Hot electrons generated from plasmonic Au species were injected into the Pt species. The O_2_ molecules adsorbed on the Pt surface could receive a hot electron to produce freestanding superoxide radicals (O_2_^•−^), which are responsible for the catalytic oxidation of the reactant. For AuPt@HMZS catalysts, there is no doubt that O_2_ molecules need to diffuse into the confined space through channels in the shell. Therefore, accumulation and collision of reactants in the confined space also accelerate the reaction (Fig. 1c).

This study provides a novel avenue for design of high-performance and sustainable photocatalysts for efficient chemical synthesis. The role of confined space will be certainly a good research direction, especially in light absorption, heat management, substrate accumulation, and protection of active components. Complex and hierarchical hollow structures, such as hollow multishelled structures (HoMSs), may provide more opportunities for rational design of efficient catalysts. The development of new technology to monitor the confined space parameters of nanoreactors renders visible the ‘black boxes’ of reactions occurring in confined space.

## FUNDING

This work was supported by the Program for Changjiang Scholars and Innovative Research Team in University (IRT 15R52) of the Chinese Ministry of Education, the National Natural Science Foundation of China (NSFC-U1663225), the Major Programs of Technical Innovation and the Overseas Expertise Introduction Project for Discipline Innovation (111 Project) (B20002).

***Conflict of interest statement*.** None declared.
